# Cell Therapy With TILs: Training and Taming T Cells to Fight Cancer

**DOI:** 10.3389/fimmu.2021.690499

**Published:** 2021-06-01

**Authors:** Amrendra Kumar, Reese Watkins, Anna E. Vilgelm

**Affiliations:** ^1^ Department of Pathology, The Ohio State University, Columbus, OH, United States; ^2^ The Arthur G. James Cancer Hospital and Richard J. Solove Research Institute, The Ohio State University, Columbus, OH, United States

**Keywords:** tumor infiltrating lymphocyte (TIL), cell therapy, immunotherapy, response biomarkers, toxicity, adoptive cell therapy (ACT), T-lymphocytes

## Abstract

The rationale behind cancer immunotherapy is based on the unequivocal demonstration that the immune system plays an important role in limiting cancer initiation and progression. Adoptive cell therapy (ACT) is a form of cancer immunotherapy that utilizes a patient’s own immune cells to find and eliminate tumor cells, however, donor immune cells can also be employed in some cases. Here, we focus on T lymphocyte (T cell)-based cancer immunotherapies that have gained significant attention after initial discoveries that graft-versus-tumor responses were mediated by T cells. Accumulating knowledge of T cell development and function coupled with advancements in genetics and data science has enabled the use of a patient’s own (autologous) T cells for ACT (TIL ACTs). In TIL ACT, tumor-infiltrating lymphocytes (TILs) are collected from resected tumor material, enhanced and expanded *ex-vivo*, and delivered back to the patient as therapeutic agents. ACT with TILs has been shown to cause objective tumor regression in several types of cancers including melanoma, cervical squamous cell carcinoma, and cholangiocarcinoma. In this review, we provide a brief history of TIL ACT and discuss the current state of TIL ACT clinical development in solid tumors. We also discuss the niche of TIL ACT in the current cancer therapy landscape and potential strategies for patient selection.

## Introduction

Cancers develop within the complex tissue microenvironment consisting of diverse cells including innate and adaptive immune cells. There are dynamic interactions between tumor cells and cells of the immune system. Effective anti-tumor immunity leads to tumor clearance, however, in certain instances, tumor cells develop strategies to evade tumor immunosurveillance and multiply uncontrolled (1–7).

Adoptive cell transfer (ACT) is a cell-based therapy that uses either the patient’s own (autologous transfer) or a donor’s (allogeneic transfer) immune cells to improve immune function. Immune cells can be modified and/or expanded *ex vivo* before they are infused back into patients as therapeutic agents. In cancer therapy, ACT strategies have been developed to overcome hypo-responsiveness of the immune system to the tumor and to boost anti-tumor immunity. Immune subtypes delivered by ACT can include dendritic cell (DC)-, natural killer (NK)-, and T lymphocyte (T)- cell-based immunotherapies, each of which is at various stages of pre-clinical and clinical development (8–18). Here, we will focus on T cell based ACT wherein a patient’s tumor-infiltrating T cells (TILs) are manipulated *ex vivo* and re-infused back into the patient.

## History of TIL ACT

### Premise

**Figure 1:**
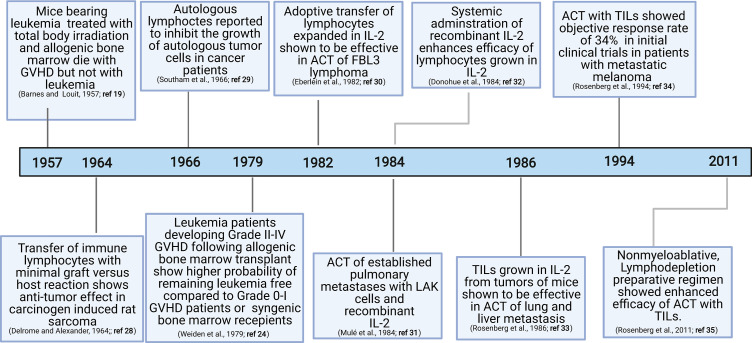
Landmark discoveries that aided the development of T cell based ACTs.

The discovery of graft-versus-tumor responses (19–25) and subsequent demonstration of the key role played by T cells in this process have motivated exploration of the role of T cells in anti-tumor immunity (26, 27) (**Figure 1**). Since graft-versus-tumor responses were often accompanied by dominant graft-versus-host disease (GVHD) in experimental models, direct assessment of graft-versus-tumor responses were not initially feasible in humans. The landmark achievement in the field that demonstrated the viability of adoptive transfer was the discovery that immune lymphocytes could treat primary fibrosarcoma in rats (28) and autologous or allogenic leukocytes could generate potent anti-tumor responses in human studies (24, 29).

### IL-2

Rosenberg and colleagues discovered that lymphocytes grown in the presence of IL-2 were capable of lysing fresh syngeneic or autologous tumors while sparing normal cells (36–40). In addition, experimental models demonstrated that either high dose injection of IL-2 alone, adoptive transfer of *ex vivo* cultured lymphocytes, or concurrent IL-2 and lymphocyte administration could deliver potent anti-tumor activity (30–32, 41–45). In human studies, concurrent administration of lymphocytes raised in IL-2 and systemic IL-2 treatment resulted in complete and durable tumor regression of metastatic melanoma tumors in a subset of patients (44).

### TILs

**Figure 2:**
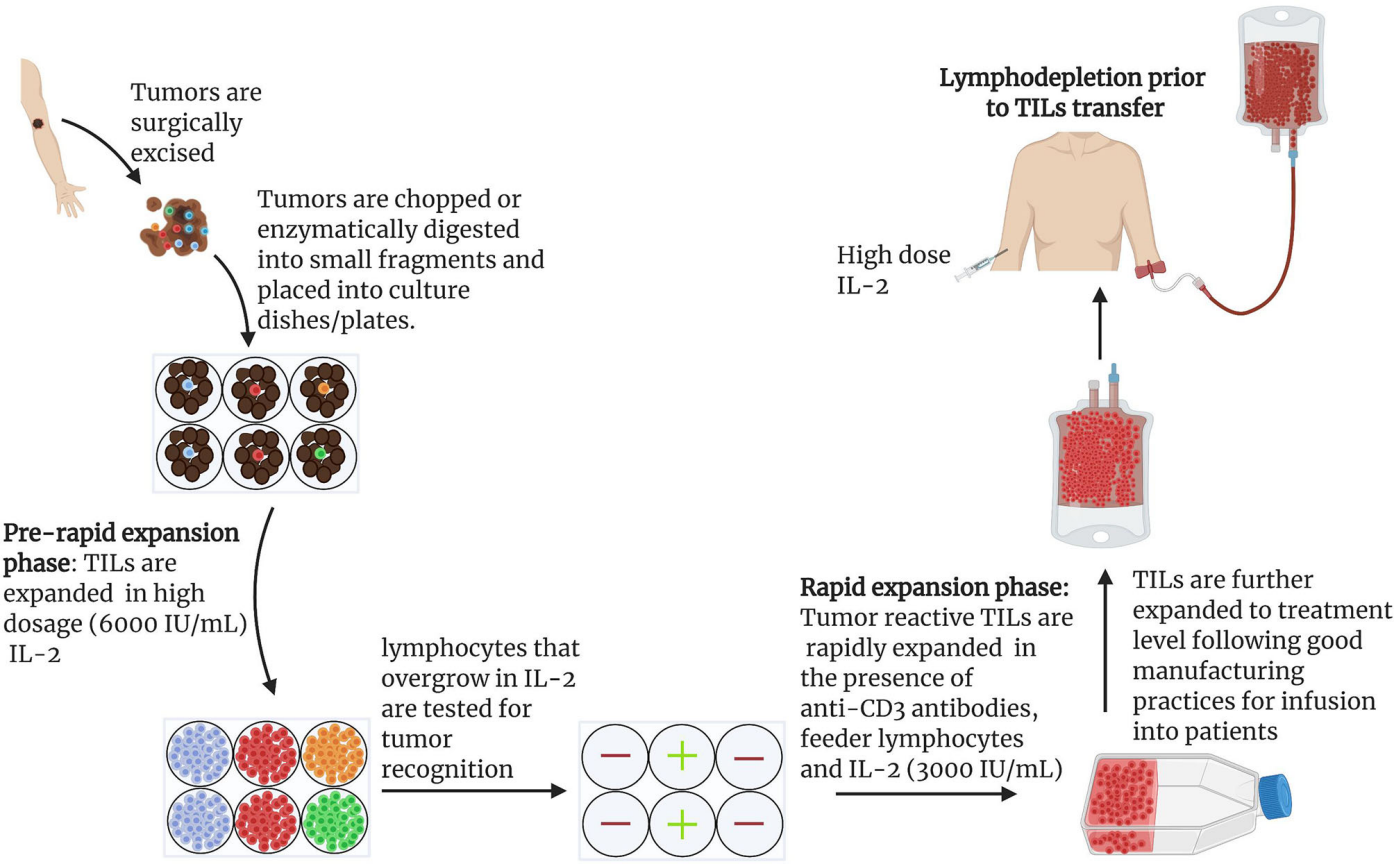
General scheme for the expansion of naturally occurring TILs for use in ACTs: Protocol for the expansion of TILs for clinical use has been described in detail (15, 47). Under anesthesia, tumors are excised from patients and cut into small pieces or digested enzymatically to obtain single cell suspensions (15, 47). Tumor fragments are grown individually in high dose IL-2 (6000 IU/mL). Under the influence of IL-2, cytotoxic lymphocytes overgrow and kill tumors within 2-3 weeks (15). Cytotoxicity of pure lymphocyte cultures are tested by co-culturing IL-2 primed lymphocytes and tumor cells. Individual cultures with high toxicity against target tumors can be rapidly expanded in the presence of irradiated feeder lymphocytes, an antibody targeting the epsilon subunit within the human CD3, and IL-2. Using this approach, Rosenberg and colleagues harvested approximately 10^11^ lymphocytes in approximately 5-6 weeks for infusion into patients. In later studies, a lymphodepletion preparative regimen consisting of 60mg/kg cyclophosphamide for 2 days and 25 mg/m^2^ fludarabine administered for 5 days demonstrated remarkable outcome in effectiveness of ACTs. Patients were infused with cells and IL-2 at 720,000 IU/kg to tolerance after lymphodepletion (15).

In early studies, T lymphocytes for ACTs were obtained from a patient’s peripheral blood by repeated lymphocytaphereses. Rosenberg’s group was the first to utilize TILs for ACT based upon the reasoning that TILs would be enriched for tumor-reactive T cells. Notably, adoptive transfer of IL-2-expanded TILs was 50 to 100 times more effective than IL-2-expanded lymphocytes from peripheral blood in mediating regression of established lung and liver tumors in mice (33). Human TILs grown from resected melanomas in the presence of recombinant IL-2 showed high potency against autologous melanomas (46). Remarkably, TILs could be expanded with high efficiency (about 95,652-fold; **Figure 2**) while maintaining robust anti-tumor cytotoxic functions (46). Using adoptive transfer of *ex vivo* generated autologous TILs, an objective response rate of 34% was observed in initial clinical trials in patients with metastatic melanoma (34). However, the median response rate was only 4 months even though several patients showed complete responses (34).

### Lymphodepletion

Another cornerstone event in the T cell ACT history was the discovery that lymphodepletion can provide a substantial increase in the persistence of transferred T cells *in vivo*. Specifically, a lymphodepletion preparative regimen consisting of 60mg/kg of cyclophosphamide for 2 days and 25 mg/m^2^ of fludarabine administered for 5 days prior to ACT increased both the rate and the duration of clinical response in patients with metastatic melanoma (**Figure 2**) (35). Among 93 patients recruited, 20 (22%) exhibited complete tumor regression, 19 of whom were in complete remission 3 years after treatment (35).

The cellular and molecular mechanisms whereby lymphodepletion regimens enhanced functions of infused TILs are still being sought out. In mouse models and in humans, lymphodepletion prior to cell transfer showed manifold improvement in the effectiveness of ACTs through enhanced persistence of transferred cells (48). Studies incorporating the lymphodepletion regimen documented enriched CD8 T cells in the patient’s peripheral blood (48, 49). Lymphodepletion has been shown to induce cytokines IL-7 and IL-15, both of which are involved in homeostasis of T cells and promote the expansion of transferred T cells in the absence of endogenous lymphocytes (50, 51). Insights gained from mouse studies suggest that lymphodepletion could enhance the efficacy of TILs through elimination of immunosuppressive cells such as myeloid derived suppressor cells (MDSCs) and FoxP3^+^ regulatory T cells (Tregs) (52, 53). In human studies and clinical studies, reappearance of FoxP3^+^ inhibitory T cells after lymphodepletion was inversely correlated with clinical response to ACTs (53). Lymphodepletion regimens have also been proposed to provide microbial-derived adjuvants, such as toll-like receptor ligands, through mobilization of microbiota and persistence and expansion of both TILs (54) and T cells engineered with chimeric antigen receptor (CAR-T cells) (55). Gaining further mechanistic insights will lead to better combinatorial approaches with ACTs to induce robust tumor-specific immunity.

## Considerations of TIL Composition

It has been recently shown that the composition and the phenotypes of TILs used for ACT play an important role in determining the therapeutic outcome. In cancer immunotherapy, both CD8 and CD4 T cells play a role in tumor rejection, although, the field has focused more on understanding anti-tumor cytotoxicity mediated by CD8 T cells (56–59). For instance, MHC class 2 (MHCII) antigen HLA-DQ O6-restricted CD4 T cells, which recognize the ERBB2IP mutation, were identified in TIL cultures from a patient with cholangiocarcinoma. While bulk TILs did not show any objective clinical response, TILs enriched to contain more than 95% ERBB2IP mutation-reactive CD4 T cells induced dramatic regression of liver and lung metastasis (56). Similarly, adoptive transfer of interleukin-17 (IL-17) producing T helper 17 (Th17) cells was shown to induce durable anti-tumor immunity suggesting that polarization of CD4 T cells plays an important role in determining anti-tumor immunity (60). Also, long term response in a patient adoptively transferred with TILs correlated to expansion and persistence of CD4 T cells directed against tumor specific mutation BRAFV600E (58). Additional evidences highlight the key roles played by CD4 T cells beyond their role in providing help to CD8 T cells. When CD8 T cells were depleted from melanoma, TILs and the remaining T cells (median 89% CD4 T cell composition assessed by flow cytometry) were assayed for tumor reactivity; about 20% showed tumor reactivity as assessed by IFNγ production (61). Blocking MHC class 2 (MHCII) with anti-HLA-DR antibodies specifically blocked IFNγ production suggesting these responses were generated by CD4 T cells (61). More recently, lung TILs expanded from NSCLC patients were found to consist of a higher number of CD4 T cells when compared to melanoma TILs that are enriched for CD8 TILs (62). Overall, these data suggest that inclusion of CD4 and CD8 T cells that recognize tumor antigens presented by major histocompatibility class II (MHCII) and MHCI may be a good therapeutic strategy to generate effective TIL ACT products, which is a term for expanded and activated TILs ready for infusion into a patient.

## TIL Selection Based on Cell Phenotype

Only a fraction of TILs (approximately 30%) are tumor reactive. Selecting for tumor reactive TILs can significantly reduce culture time and minimize the number of infused cells. Expression of PD-1 was found to be high on melanoma reactive TILs and PD-1 positive TILs showed enhanced tumor reactivity compared to PD-1 negative TILs (63). Expression of CD137/4-1BB, a CD8 T cell activation marker, was used to select tumor reactive TILs from melanoma patients. Bead sorted or FACS sorted CD137 cells showed enhanced tumor reactivity compared to unselected TILs (64). Recently, sorting and expansion of CD137 TILs has been achieved in a large scale manner meeting GMP requirements for infusion into patients (65). Also, CD8+PD-1+CD103+ tissue-resident memory (TRM) T cells subpopulation are associated with better survival outcomes in many solid tumors owing to their enhanced capacity to home to tumors due to the expression of integrins on their cell surface (66–68). A subset of exhausted T cells known to express the transcription factor TCF1 are known to possess self-renewing properties and long term maintenance of persistent T-cell responses in different solid tumors (69–73). Also, there are efforts to isolate tumor reactive T cells with high expression of co-stimulatory molecules such 4-1BB or OX-40 or metabolically “fit” T cells as it is expected to reduce loss of anti-tumor function during expansion in IL-2 culture conditions (57, 74–76). In addition to the inclusion of diverse T cell subsets, another factor associated with TIL persistence is the presence of stem-like cells. A recent study demonstrated that a minor population of a memory-progenitor CD8 T cell with stem-like phenotype (CD39^-^CD69^-^) is responsible for complete cancer regression and TIL persistence. The authors linked the superior responses to ACT containing stem-like T cells with their ability to self-renew *in vivo* (73).

## Tailoring TIL ACT to Target Tumor Antigens

Unselected TIL products are comprised of tumor-derived T cells with diverse specificities. While in comparison to circulating T cells TILs are enriched with tumor-specific T cells, they also contain non-specific cells. As a result, not all T cells within TIL ACT products are expected to have potent tumor reactivity. This is especially relevant for tumors that are not highly distinct from the patient’s normal tissue in terms of their antigen profiles. Indeed, tumors with low levels of unique antigens are likely to contain low levels of tumor-specific TILs and, therefore, may give rise to TIL ACT products with suboptimal efficacy. Identification and selective expansion of T cells with a pre-defined specificity towards unique tumor antigens is a promising strategy to increase the chances of successful ACT in such individuals. There are several strategies that are currently in clinical development to identify tumor-associated antigens and generate personalized ACT products for superior tumor control.

### TIL ACT Targeting Tumor Neoantigens

Perhaps the most advanced strategy to personalize TIL ACT is to identify and expand TILs with TCRs specific towards tumor neoantigens. Tumors often exhibit unique alterations in their DNA such as single nucleotide changes, and insertions and deletions that accumulate in the tumors and lead to frameshift and structural variants. Neoantigens are the protein products of the altered genes that are processed and presented by MHC molecules that are capable of eliciting T cell responses [See (77, 78) for reviews]. Due to the fact that cancer neoantigens constitute “non-self”, T cells recognizing neoantigens that arise in the thymus will escape host central tolerance mechanisms that eliminate autoreactive T cells. Since neoantigens have restricted expression in tumors, neoantigen-specific T cells are not likely to generate “on target, off tumor” toxicities. Finally, generation of neoantigen-specific T cells is a stochastic event. Not all random mutations affect protein-coding regions and not all mutated proteins can be recognized by T cells. The more somatic mutations a given tumor has, the greater the likelihood of that tumor possessing neoantigens and responding to agents that stimulate endogenous anti-tumor immunity (79–81). With an appropriate production pipeline, even a small number of T cells specific to scarce tumor neoantigens can be expanded for therapeutic application. Based on these considerations, ACT with TILs enriched with neoantigen-specific T cells is an attractive therapeutic option, especially for tumors with a low mutational burden.

Efforts to isolate neoantigen-specific T cells from TILs have gained pace after recognition that neoantigen-specific T cells play a critical role in maintaining durable responses following TIL ACT. Huang et al., found that T cells in patients that exhibited regression of multiple metastatic melanoma lesions consisted of clones that recognized frameshifted products of the tumor suppressor gene CDKN2A and a mutated HLA class I gene product (82). Zhou et al. reported recognition of mutated growth arrest-specific gene 7 (GAS7) and glyceraldehyde-3-phosphate dehydrogenase (GAPDH) gene products in another patient that showed complete regression of all metastatic lesions in lungs and soft tissues following TIL therapy (83). This study found at least six clonotypes that persisted in peripheral blood for months following therapy using direct sequencing analysis of T cell receptor beta chain (TCRb), including those recognizing GAS7 and GAPDH (83). Several other reports have emerged that demonstrated the vital contribution of tumor neoantigen-specific T cells clones in maintaining durable anti-tumor responses (84–88).

**Figure 3:**
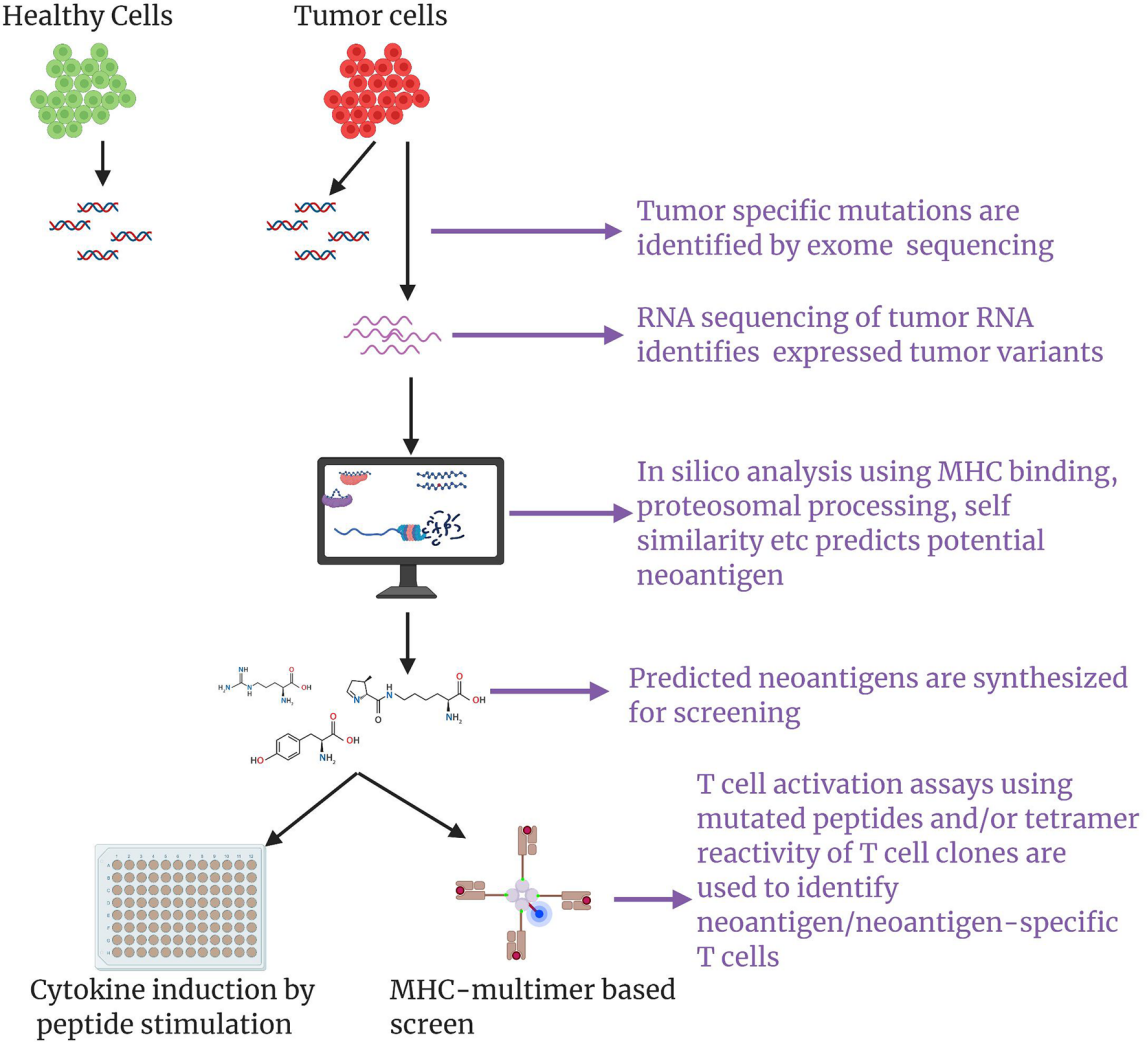
A schematic of neoantigen discovery pipeline: Using next generation exome sequencing, exome sequences of healthy cells and tumor cell are obtained and comparative analysis results in identification of tumor associated mutations. RNA sequencing of the tumor ascertains expressed tumor variants. Appropriate *in silico* methods such as prediction of peptide binding to the patient’s MHC haplotypes, peptide cleavage products generated by proteosome etc. are applied to predict putative neoantigens. Use of mass spectrometry analysis of MHC-associated peptides in tandem with an *in silico* approach could greatly aid neoantigen prediction/discovery. Putative neoantigens are synthesized and screened for eliciting neoantigen specific T cell responses through multimer based screen or cytokine induction by peptide stimulation.

Initial studies utilized autologous tumor cell cDNA libraries for screening neoantigen-specific T cells. These approaches were cumbersome and impeded the progress of neoantigen discovery and application (82, 83, 89). Later, Rosenberg and colleagues devised a conceptually new strategy outlined in **Figure 3** that did not require the laborious cDNA library screens (85). In this approach, comparative analysis of NGS exome sequencing data of tumor and healthy tissue was used for identification of mutated proteins. Expression of mutated proteins was measured by RNAseq of tumor tissue. Using a major histocompatibility complex-binding algorithm, putative T cell epitopes were identified, synthesized, and then evaluated for recognition by TILs. This filtering approach significantly reduced the number of candidate neoantigens for T cell reactivity assays (85). Several other studies also demonstrated that cancer genome data obtained from NGS exome sequencing of tumors from mice and humans could be used for discovery of putative neoantigens and as an assay of T cell reactivity against these neoantigens (77, 78, 85, 90–92). Using these approaches, neoantigens and T cell reactivity to these neoantigens have been identified in a variety of cancers including NSCLC, ovarian cancer, squamous cell carcinoma of the head and neck, cholangiocarcinoma, and colorectal cancer (56, 81, 88, 93, 94). Recently, researchers have demonstrated that tandem minigen (TMG) libraries encoding putative neoantigens could be utilized to screen for therapeutic TILs and identification of neoantigen-specific T cells (87–89).

Once neoantigen-specific T cells are identified, they can be further purified using flow cytometers (89) and expanded *ex vivo* using a strategy described in **Figure 2**. Transfer of neoantigen-specific CD4 T and CD8 T cells enriched from TIL cultures and selected on the basis of high reactivity against neoantigens, has shown complete and partial responses that were durable in patients with metastatic cholangiocarcinoma (56), colorectal cancer (93), and breast cancer (95). However, in other studies, neoantigen-specific T cells derived from TIL cultures from patients with metastatic gastrointestinal cancers have shown limited clinical responses (96).

One potential factor contributing to poor efficacy of TIL-derived neoantigen-specific T cells is the irreversible hypo-responsiveness of tumor-infiltrating T cells induced by the suppressive tumor microenvironment. An alternative strategy that has been explored to produce effective ACT therapies is to activate naïve tumor-specific T cells *ex vivo* or generate neoantigen-specific T cells de novo by means of TCR genetic engineering. The latter approach of ACT with TCR-engineered T cells has been described in detail elsewhere (18, 97, 98). An example of the first approach comes from the study by Verdegaal et al. In this study, autologous melanoma cell lines were cultured with autologous peripheral blood cells to activate and expand tumor-specific T cells (99). Analysis of infused batches of tumor-specific T cells revealed the presence of polyclonal tumor-reactive CD8 T and CD4 T cells. Out of ten patients infused with the resulting product, one experienced a complete response, one had a partial response, and three patients exhibited disease stabilization (99). This suggests that autologous PBMC could be a viable source of neoantigen-specific T cells for ACTs. Currently, efforts are being made to develop efficient strategies for the isolation of neoantigen-specific T cells and their rapid expansion that could further aid ACTs with neoantigen-specific T cells (56, 100, 101).

### TIL ACT Targeting Cancer/Testis Antigens and Proteins Overexpressed by Tumor

Another strategy explored to enhance tumor specificity of T cells ACT products is to target them towards cancer/testis antigens. Cancer/testis antigens are a group of proteins that are normally expressed in immune-privileged tissues, such as testicular germ cells and placental trophoblasts, but not in adult somatic cells. Some malignant tumors re-express cancer/testis antigens presenting an opportunity for developing immunotherapies targeting these molecules [see (102–104) for dedicated reviews]. Unlike tumor neoantigens that are unique in each patient, cancer/testis antigens are shared, offering a more streamline production process. As reviewed by Whitehurst et al, 70% of metastatic melanomas express MAGEA-1-4, 70% of ovarian tumors express ACRBP, and 46% of breast cancers express NY-ESO-1 (105). Adoptive transfer of T cells engineered to express NY-ESO-1-specific T-cell receptors have been tested in melanoma and synovial sarcoma with response rates as high as 45% and 67%, respectively (106). In addition, another study reported tumor regression in 5 out of 9 melanoma patients treated with autologous anti-MAGE-A3 TCR-engineered T cells. However, neurologic toxicity was also reported (107).

Distinct tumor types can display high levels of certain normal non-mutated proteins. For example, tumors produced by melanocytic cells, including cutaneous and uveal melanomas, express high levels of proteins involved in the melanin biosynthesis pathway, such as MART-1 and gp100. However, normal non-malignant melanocytes also highly express these proteins. Not surprisingly, clinical development of ACTs targeting melanoma lineage markers had limited success. For example, ACT with peripheral T cells engineered to express TCRs with high affinity for MART-1 and gp100 targeted not only tumor cells, but also normal melanocytes in the eye and ear. This led to uveitis and hearing loss (108). Even though the efficacy of ACT targeting melanocyte markers is promising, causing cancer regression in 30% of tested subjects, substantial on target toxicities need to be addressed for further development of these strategies. For a more detailed review on identification and immunotherapeutic targeting of diverse tumor antigens, please see the review by Leko et al. (109).

## Current State of TIL ACT Clinical Development

To date there have been many TIL ACT studies completed and some demonstrated encouraging results. Based on reported data, the most prominent anti-tumor activity of TIL ACT is seen in melanoma patients. This is well illustrated by a systematic review and meta-analysis study performed by Dafni et al. (110). Authors analyzed 13 clinical studies of TIL ACT combined with IL-2 administration, including 7 studies where high dose IL-2 was used. Studies were conducted within the 2005-2016 period. High dose IL-2 and TIL ACT produced better response rates when compared to low dose IL-2. Based on combined data from seven individual studies with 332 patients in total, the average objective response rate for TIL ACT and high IL-2 therapy was 44%. Complete responses that were durable were reported in 49 patients.

While TIL ACT has not yet received an FDA approval for the treatment of solid tumors, it is in active clinical development. A number of clinical trials have reported encouraging results. The commercial autologous TIL product lifileucel (LN-145, LN-144 and LN-145-S1), developed by Iovance Biotherapeutics, is in phase II clinical development for patients with unresectable or metastatic melanoma, recurrent or metastatic head and neck squamous cell carcinoma (HNSCC), recurrent or metastatic non-small cell lung cancer (NSCLC), or relapsed or refractory NSCLC. The manufacturing process disclosed by the company is relatively simple. Surgically resected tumor cells are shipped to the central manufacturing facility where they are fragmented and cultured in the presence of IL-2 to allow TIL to egress from the tumor and expand to approximately 10^9^–10^11^ cells per culture. Next, the TIL are washed, placed in the infusion bags and cryopreserved. The data from 66 heavily pretreated melanoma patients were presented at the 2020 American Society of Clinical Oncology (ASCO) Annual Meeting. The study recruited patients that progressed on multiple prior therapies including immune checkpoint blockade and BRAF inhibition, and who also exhibited high tumor burden, with many having liver and brain lesions. Remarkably, an overall response rate of 36.4% was achieved after a single infusion of lifileucel along with lymphodepletion and an IL-2 regimen (111). To put this into perspective, chemotherapy is the only treatment option currently available for the patient population enrolled in this trial. Chemotherapy is effective only in 10% of patients, and responses are typically of short duration.

Another TIL product developed by Iovance, LN-145, demonstrated promising preliminary efficacy results in 27 patients with advanced cervical cancer who have undergone at least one prior line of chemotherapy. As per a report at ASCO 2019, there was a 44% objective response rate observed, which included 1 complete response and 9 partial responses. In contrast, the objective response rate of approved second line chemo- and immunotherapies for these patients falls within the 4-14% range (112). Not surprisingly, the FDA has granted Breakthrough Therapy designation to LN-145 in recurrent, metastatic, or persistent cervical cancer with disease progression on or after chemotherapy. Breakthrough Therapy designation is designed to expedite the development of emerging therapeutics in the case where preliminary clinical evidence indicates that the drug may offer substantial improvement over available therapies. If the preliminary efficacy of lifileucel and LN-145 holds in larger cohorts and the responses are durable, we may expect an FDA approval of TIL ACT for solid tumors in the near future.

There is some clinical evidence suggesting that TIL ACT may be effective in patients with tumors other than melanoma and cervical cancer. Preliminary results of a phase I clinical trial that evaluated TIL ACT for metastatic NSCLC were very encouraging (113). In this study TILs were successfully expanded in 95% of patients. A total of 20 patients were enrolled and 13 of them showed evidence of progression on nivolumab therapy. Patients received cyclophosphamide/fludarabine lymphodepletion therapy followed by ACT with TIL and IL-2. Tumor regression was noted in a majority of patients after administration of TILs (median time-on-trial post TIL was 1.4 years). Two patients achieved durable clinical responses which were ongoing 1-year post-TIL administration.

In addition, case reports indicated durable remission after TIL ACT in patients with metastatic breast cancer (95), metastatic colorectal cancer (93), and metastatic cholangiocarcinoma (56). There are many TIL ACT clinical studies recruiting patients as of 03/12/2021, including trials in biliary tract cancers (NCT03801083), metastatic uveal melanoma (NCT03467516), gynecologic tumors (NCT04766320), pretreated metastatic triple negative breast cancer (NCT04111510), non-small-cell lung cancer (NCT04614103), colorectal cancer (NCT03904537), ovarian cancer (NCT04072263), cervical carcinoma (NCT04443296), relapsed or refractory ovarian cancer, anaplastic thyroid cancer, osteosarcoma, or other bone and soft tissue sarcomas (NCT03449108), and others.

## The Niche for TIL ACT in the Current Landscape of Cancer Immunotherapy

**Figure 4:**
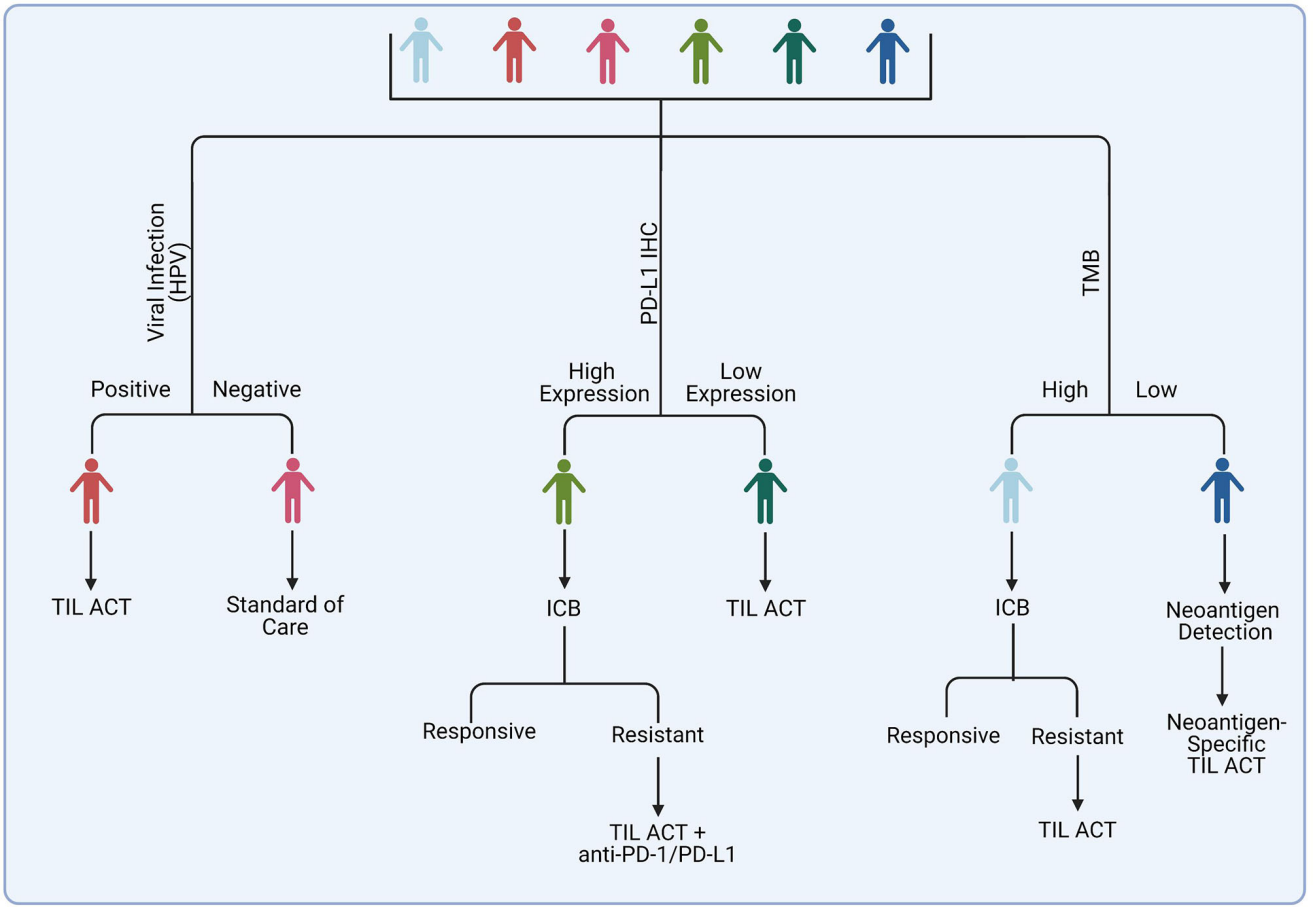
Potential strategy for selecting patients for TIL ACT administration. Among suggested candidates are patients with virally-infected tumors and those who acquired resistance on ICB therapy. Tumors with a high mutational burden may respond to ACT with unselected TIL, whereas tumors with poorly immunogenic tumor may benefit from identification of specific tumor neoantigens and generation of a specialized TIL-ACT products targeting those neoantigens.

Currently, there are pivotal TIL ACT trials ongoing in melanoma and cervical cancer. The results of these trials will guide the FDA decision on the approval of this treatment for clinical use. If we are to keep an optimistic outlook, it may be a good time to think about how TIL ACT will fit into the current cancer treatment toolset (**Figure 4**).

The field of solid tumor immunotherapy is dominated by the immune checkpoint blockade (ICB) agents. Melanomas are among the malignancies that are most responsive to ICB. ICB therapies stimulate anti-tumor T lymphocytes by blocking the interactions between inhibitory immune checkpoint ligands and their receptors, such as CTLA-4 and PD-L1 (114). The rate of response to anti-PD-1 therapy alone is about 40% and about 60% for anti-PD-1 and anti-CTLA4 combined. For instance, in the phase III CheckMate 067, the rate of objective response was 43.7% for nivolumab alone and 57.6% for nivolumab plus ipilimumab (93). In many cases the responses are durable. The ICB therapy can induce severe toxicities in some patients, however, the understanding and management of these toxicities has significantly improved since these drugs have been used to treat cancer patients for over a decade. The average reported response rates to TIL ACT in melanoma is 43% (110), which is comparable to anti-PD-1 but lower than that with anti-PD1 and anti-CTLA4 combined. There are also logistical challenges associated with TIL ACT that do not compare favorably to ICB. ICB agents are universal and ubiquitously available. Therefore, patients can begin treatment as soon as there is a medical need for it. In contrast, TIL products need to be custom made for an individual patient. This process requires a specialized GMP-compliant facility, skilled personnel, and it takes time. Currently, the fastest TIL production of 22 days has been achieved by Iovance. Other groups report a minimum 6-8-week production time. This time can be significantly longer if selection of tumor-specific or tumor-neoantigen-specific TILs is performed. This would be an issue for patients with aggressive metastatic cancers that may not have much time to spare waiting for a custom therapy. Furthermore, the costs of TIL ACT significantly exceed that of ICB.

Considering the aforementioned factors, one rational option is to use TIL ACT as a second line therapy in patients who received ICB and were not responsive or acquired resistance. The success of this approach was demonstrated by a number of trials in melanoma patients heavily pre-treated with immunotherapy and other therapies where objective and often durable responses were achieved in about 20-30% of patients (35, 110, 115–117).

Another potential opportunity for introducing TIL ACT into the clinic is to offer it to patients who are not likely to respond to immune checkpoint blockade. There has been tremendous progress in the field of ICB response biomarkers in recent years, fueled by a growing pool of clinical specimens from patients treated with ICB, advancements in –omics technologies, and the increase of computational power and machine learning capabilities necessary for mining high content data. As a result, clinicians can predict to some extent whether or not a given patient is likely to respond to ICB therapy based on analysis of tumor and/or non-tumor markers. One important question is whether the same mechanisms that allow tumor cells to overcome the ICB-induced immune response driven by endogenous T cells, will also facilitate resistance to TIL ACT. In the section below, we review key factors of ICB response and resistance and discuss how they may influence TIL ACT outcome.

## Potential Biomarkers for Selecting Patients for TIL ACT

### PD-L1 Immunohistochemical Staining

The T cell inhibitory molecules PD-1 and PD-L1 have emerged as the frontrunners among cancer immunotherapies that have been approved for clinical use. The interaction between programmed death ligand-1 (PD-L1) and its receptor PD-1 functions in immune self-tolerance under homeostatic conditions. However, it is also common for the tumor microenvironment (TME) to be enriched in PD-L1 to promote tumor tolerance by the immune system. Overexpression of PD-L1 has been proven to inhibit the T cell-mediated anti-tumor immune response, allowing the tumor to evade immunity. Consequently, several biologic therapies targeting the interaction of PD-L1 with PD-1 have been developed. Since high levels of PD-L1 indicates an active state of the PD-1/PD-L1 checkpoint in the tumor, expression of PD-L1 by the tumor was one of the first and most extensively investigated candidate biomarkers for predicting the outcome of PD-1/PD-L1 targeting immunotherapy (118). Positive immunohistochemical (IHC) staining against PD-L1 has been linked with increased clinical responsiveness to anti-PD-1 and anti-PD-L1 therapy in select tumor types. Based on these findings, PD-L1 IHC has been approved for clinical use as a predictor of anti-PD-L1 treatment efficacy in NSCLC (119).

While a positive response biomarker for anti-PD1 therapy, high levels of tumor PD-L1 are likely to inhibit the activity of transferred TILs within the TME via interactions with the PD-1 receptor. One potential way to overcome this obstacle is to combine TIL ACT with anti-PD1 therapy. There are a number of clinical trials ongoing testing this hypothesis. In a recent study, six patients with late-stage metastatic high-grade serous ovarian cancer were treated with ipilimumab followed by surgery to obtain TILs. Patients then received TILs with low-dose IL-2 and nivolumab. One patient showed a partial response and 5 others exhibited disease stabilization (120). 

### Tumor Mutational Burden

There is a connection between the abundance of tumor somatic mutations and ICB response. Genetically altered genes can produce mutated proteins that can be recognized by the immune system as “non-self” when processed and presented on the cell surface by the MHC molecules (77, 78, 121). The tumor mutational burden (TMB) and subsequent probability of high neoantigen content differs significantly between distinct cancer types. Tumors that are induced by external carcinogen exposure, such as UV radiation in melanoma and smoking in lung cancer, tend to have high mutational burdens (122) and, therefore, are predicted to be more immunogenic as there are more potential targets for T cells to respond to (123). Several retrospective studies have linked a high TMB with responsiveness to PD-1 inhibition (81, 124). A recent prospective clinical study, Keynote-158, demonstrated that patients with high TMB were more likely to respond to anti-PD-1 agent pembrolizumab across 10 distinct solid tumor types (125). Based on these results, the FDA has approved the use of pembrolizumab for patients with high TMB (defined as having ≥10 mutations/megabase) regardless of tumor type (FDA approves pembrolizumab for adults and children with TMB-H solid tumors. News release. FDA. June 17, 2020. https://bit.ly/30QEt40]).

Of note, tumors with deficiencies in the mismatch repair (MMR) pathway are likely to contain high levels of genetic mutations, thereby increasing the probability of expressed neoantigens (126). Clinical studies have demonstrated that colorectal cancer patients with an impaired MMR pathway are significantly more likely to respond to ICB as compared to patients with MMR-proficient tumors (127, 128).

Tumors with high neoantigen content are more likely to have high levels of tumor-reactive TILs. There is an inter-clonal competition ongoing during the expansion of TILs. The presence of a relatively high number of tumor-specific T cell clones in the starting culture is likely to ensure a sufficient number of tumor-reactive TILs in the final ACT product. Also, TILs with reactivity against multiple epitopes would ensure that loss of any specific antigen does not subvert the clinical anti-tumor responses. Not surprisingly, TIL ACT has shown reproducible efficacy in patients with melanoma tumors that commonly exhibit a high TMB (**Table 1**). While there is a link between high mutation content and response to TIL ACT in its standard form, there is a hope for tumors with a relatively low abundance of mutations offered by an ACT with TILs engineered to target specific tumor neoantigens. With the tremendous advancements in our ability to identify potent neoantigens capable of inducing strong immune responses and to generate T cells with corresponding specificity, even rare mutational events can be targeted with precision and efficiency.

**Table 1:** Selected clinical trials of TIL ACT that reported results.

Cancer type	Patient cohort	Therapeutic agents	Objective response rate (responders/total n)	Complete response rate(responders/total n)	Ref.
Metastatic melanoma (meta-analysis of 7 studies)		TIL	43% (141/332)	15% (49/332)	(110)
Metastatic melanoma	Bulky tumor, multiple prior therapies, progression on ICB	Lifileucel	36.4% (24/66)	3% (2/66)	(111)
Cervical cancer		TIL + Anti-PD-L1	25% (20/80) HPV+: 20/68 HPV-: 0/12	5% (4/80)	(129)
Cervical cancer	HPV+	HPV-specific TIL	28% (5/18)	11% (2/18)	(130)
Cervical cancer	Advanced cancer with at least one prior therapy	LN-145	44% (12/27)	4% (1/27)	(112)
NSCLC	Progression after nivolumab alone	TIL and nivolumab	Not specified	10% (2/20)	(113)

### Viral Infections

Neoantigens are not the only source of tumor immunogenicity. The immune system has evolved to detect viral antigens, therefore virally-infected cancers, such as those associated with human-papillomavirus (HPV) or Epstein-Barr virus (EBV), can trigger T cell-mediated immunity (131). Clinical studies have shown that patients presenting with head and neck squamous cell carcinoma who were positive for HPV showed an increased response to anti-PD-1 and anti-PD-L1 inhibitors, pembrolizumab and durvalumab, respectively, when compared to HPV-negative counterparts (132, 133). Moreover, increased ICB efficacy was noted in patients with metastatic gastric cancer who were positive for EBV and HIV (134, 135).

Similar to ICB, TIL ACT tends to be effective against tumors containing viral antigens. It has been reported that patients with HPV-positive metastatic cervical cancer were significantly more likely to respond to TIL ACT as compared to HPV-negative patients (129). In another study, three out of nine cervical cancer patients responded to treatment with TILs that were pre-selected for reactivity to HPV (136). In a later study by the same group testing the efficacy of HPV-specific TIL ACT in patients with any HPV-associated epithelial cancers, the objective response rate was 28% (5 out of 18 patients) in the cervical cancer cohort and 18% (2 of 11) in the noncervical cancer cohort (130).

In addition to clinically-approved predictors of ICB sensitivity such as a high TMB, impaired MMR pathway, and PD-L1 IHC positivity, many more emerging biomarkers are currently being evaluated in preclinical and clinical settings. These include various tumor expression signatures such as an interferon signature (137), increased leukocyte infiltration (138), and the presence of inhibitory immune subsets, such as myeloid-derived suppressor cells (MDSC) and tumor-associated macrophages (TAM). Further, non-tumor markers, including gut microbiome composition and abundance of certain blood cells and proteins, have been shown to modulate anti-tumor immunity and ICB response. It is plausible that these factors may affect TIL ACT efficacy. For instance, a TME enriched with interferons and products of the interferon signature genes may enhance activity of transferred effectors, while MDSCs and TAMs are likely to limit TIL efficacy. More research is needed to validate potential predictive biomarkers that may help to identify candidates for TIL ACT.

## Common Toxicities of TIL ACT

Toxicity is always a concern with any emerging therapeutic. Based on the safety data from early phase clinical studies, TIL ACT has a relatively good safety profile. Often times, side effects are associated with co-treatments administered in conjunction with TILs, such as IL-2 and the chemotherapy regimen (139). Toxicities can be observed immediately, or they can have delayed onset. Virtually all patients undergoing non-myeloablative lymphodepleting chemotherapy experience cytopenia including neutropenia, lymphopenia, as well as prolonged depression of CD4 T cells (50, 140–146). These hemotological side effects are managed following standard good clinical practices (139, 147). Patients are treated with granulocyte-colony stimulating factor (G-CSF) and transfusions of blood-derived products (50, 140–146). Non-hematological toxicities associated with a lymphodepletion regimen include diarrhea, hyperbilirubinemia, and fludarabine-induced neurotoxicity (140, 141, 143). A minority of patients can develop opportunistic infections such as *Pneumocystis jirovecii* pneumonia or *Herpes zoster* re-activation that are controlled by routine prophylaxis treatment post chemotherapy (140). High-grade toxicities associated with TIL infusion products are very rare and often difficult to differentiate from reactions associated with residual IL-2 in the TIL products themselves (50, 140, 146, 148). Acute cytokine release with fever, skin reaction, and dyspnea are common allergic reactions (140, 149). Autoimmune reactions are mostly related to melanocyte destruction and manifest as vitiligo or uveitis in 35% and 15% cases, respectively (140, 149). High dose of IL-2 is associated with transient toxicities that can be managed with standard interventions. With adoption of a lymphodepletion regimen, IL-2 toxicities have been greatly limited since lymphocytes in immunocompetent individuals are a major sources of cytokines that contribute to IL-2-associated side effects (150). Several organs such as the heart, lungs, kidneys, and central nervous system can be affected by IL-2 toxicity. Clinicians have gained experience globally in managing toxicities associated with IL-2 and standardized guidelines have emerged (151–154). In summary, there are several toxicities associated with TIL therapy, a majority of which are low grade and manageable with standard supportive care. Specialized care centers are required for TIL therapy for managing associated toxicities (139).

## Concluding Remarks

TIL ACT is a promising emerging immunotherapy for solid tumors that is likely to be implemented into clinical practice in the near future. The undeniable advantages of TIL ACT are a) robust and reproducible clinical responses and b) the ability to benefit heavily pre-treated patients with advanced tumors who have run out of other therapeutic options. However, there are a number of challenges associated with the production and delivery of these therapies. TIL ACT is the ultimate personalized treatment since a specific infusion product has to be manufactured for every individual patient. This requires highly specialized good manufacturing practice (GMP) facilities and a trained staff, leading to high costs. Furthermore, the production process takes time, often more than a month, which can be too long for patients with rapidly progressing tumors. Commercialization of TIL ACT and streamlining of the manufacturing process are gradually addressing the logistical challenges of TIL ACT to enable wide clinical application of this promising therapeutic modality.

## Author Contributions

AK, RW, and AV wrote the manuscript. AV conserved the manuscript. AK and RW prepared figures. All authors contributed to the article and approved the submitted version.

## Funding

This work was supported by grants from NIH (R37 CA233770-01 to AV), and BCRF (IIDRP-16-001 to AV). AV is supported by Pelotonoa, OSUCCC Drug Development Institute (DDI), and Translational Therapeutics Program.

## Conflict of Interest

The authors declare that the research was conducted in the absence of any commercial or financial relationships that could be construed as a potential conflict of interest.

## References

[1] Balkwill F Mantovani A. Inflammation and Cancer: Back to Virchow? Lancet (2001) 357:539–45. 10.1016/S0140-6736(00)04046-0 11229684

[2] Dunn GP Old LJ Schreiber RD. The Three Es of Cancer Immunoediting. Annu Rev Immunol (2004) 22:329–60. 10.1146/annurev.immunol.22.012703.104803 15032581

[3] Gonzalez H Hagerling C Werb Z. Roles of the Immune System in Cancer: From Tumor Initiation to Metastatic Progression. Genes Dev (2018) 32:1267–84. 10.1101/gad.314617.118 PMC6169832 30275043

[4] Kaplan DH Shankaran V Dighe AS Stockert E Aguet M Old LJ. Demonstration of an Interferon Gamma-Dependent Tumor Surveillance System in Immunocompetent Mice. Proc Natl Acad Sci USA (1998) 95:7556–61. 10.1073/pnas.95.13.7556 PMC22681 9636188

[5] Schreiber RD Old LJ Smyth MJ. Cancer Immunoediting: Integrating Immunity’s Roles in Cancer Suppression and Promotion. Science (2011) 331:1565–70. 10.1126/science.1203486 21436444

[6] Shankaran V Ikeda H Bruce AT White JM Swanson PE Old LJ. IFNγ and Lymphocytes Prevent Primary Tumour Development and Shape Tumour Immunogenicity. Nature (2001) 410:1107–11. 10.1038/35074122 11323675

[7] Vesely MD Kershaw MH Schreiber RD Smyth MJ. Natural Innate and Adaptive Immunity to Cancer. Annu Rev Immunol (2011) 29:235–71. 10.1146/annurev-immunol-031210-101324 21219185

[8] Patel S Burga RA Powell AB Chorvinsky EA Hoq N McCormack SE. Beyond Car T Cells: Other Cell-Based Immunotherapeutic Strategies Against Cancer. Front Oncol (2019) 9:196. 10.3389/fonc.2019.00196 31024832 PMC6467966

[9] Janikashvili N Larmonier N Katsanis E. Personalized Dendritic Cell-Based Tumor Immunotherapy. Immunotherapy (2010) 2:57–68. 10.2217/imt.09.78 20161666 PMC2819192

[10] Palucka K Banchereau J. Cancer Immunotherapy Via Dendritic Cells. Nat Rev Cancer (2012) 12:265–77. 10.1038/nrc3258 PMC3433802 22437871

[11] Anguille S Smits EL Lion E van Tendeloo VF Berneman ZN. Clinical Use of Dendritic Cells for Cancer Therapy. Lancet Oncol (2014) 15:e257–267. 10.1016/S1470-2045(13)70585-0 24872109

[12] Tacken PJ de Vries IJ Torensma R Figdor CG. Dendritic-Cell Immunotherapy: From Ex Vivo Loading to In Vivo Targeting. Nat Rev Immunol (2007) 7:790–802. 10.1038/nri2173 17853902

[13] Salagianni M Baxevanis CN Papamichail M Perez SA. New Insights Into the Role of NK Cells in Cancer Immunotherapy. Oncoimmunology (2012) 1:205–7. 10.4161/onci.1.2.18398 PMC3376993 22720243

[14] Bald T Krummel MF Smyth MJ Barry KC. The NK Cell-Cancer Cycle: Advances and New Challenges in NK Cell-Based Immunotherapies. Nat Immunol (2020) 21:835–47. 10.1038/s41590-020-0728-z PMC8406687 32690952

[15] Rosenberg SA Restifo NP. Adoptive Cell Transfer as Personalized Immunotherapy for Human Cancer. Science (2015) 348:62–8. 10.1126/science.aaa4967 PMC6295668 25838374

[16] Guedan S Ruella M June CH. Emerging Cellular Therapies for Cancer. Annu Rev Immunol (2019) 37:145–71. 10.1146/annurev-immunol-042718-041407 PMC7399614 30526160

[17] Rohaan MW Wilgenhof S Haanen J. Adoptive Cellular Therapies: The Current Landscape. Virchows Arch (2019) 474:449–61. 10.1007/s00428-018-2484-0 PMC6447513 30470934

[18] Waldman AD Fritz JM Lenardo MJ. A Guide to Cancer Immunotherapy: From T Cell Basic Science to Clinical Practice. Nat Rev Immunol (2020) 20(11):651–68. 10.1038/s41577-020-0306-5 PMC7238960 32433532

[19] Barnes DW Loutit JF. Treatment of Murine Leukaemia With X-Rays and Homologous Bone Marrow. II. Br J Haematol (1957) 3:241–52. 10.1111/j.1365-2141.1957.tb05793.x 13460193

[20] De Vries MJ Vos O. Treatment of Mouse Lymphosarcoma by Total-Body X-Irradiation and by Injection of Bone Marrow and Lymph-Node Cells. J Natl Cancer Inst (1958) 21:1117–29. 13611536

[21] Bortin MM Rimm AA Saltzstein EC Rodey GE. Graft Versus Leukemia. 3. Apparent Independent Anthost and Antileukemia Activity of Transplanted Immunocompetent Cells. Transplantation (1973) 16:182–8. 10.1097/00007890-197309000-00004 4147030

[22] Bortin MM Rimm AA Rose WC Saltzstein EC Graft-versus-leukemia V. Absence of Antileukemic Effect Using Allogeneic h-2-Identical Immunocompetent Cells. Transplantation (1974) 18:280–3. 10.1097/00007890-197409000-00012 4154022

[23] Boranić M. Transplantability of Leukaemia From Leukaemic Mice After Irradiation and Injection of Allogeneic Spleen Cells. Rev Eur Etud Clin Biol (1970) 15:104–9. 4909999

[24] Weiden PL Flournoy N Thomas ED Prentice R Fefer A Buckner CD. Antileukemic Effect of Graft-Versus-Host Disease in Human Recipients of Allogeneic-Marrow Grafts. N Engl J Med (1979) 300:1068–73. 10.1056/NEJM197905103001902 34792

[25] Thomas ED Buckner CD Banaji M Clift RA Fefer A Flournoy N. One Hundred Patients With Acute Leukemia Treated by Chemotherapy, Total Body Irradiation, and Allogeneic Marrow Transplantation. Blood (1977) 49:511–33. 10.1182/blood.V49.4.511.bloodjournal494511 14751

[26] Bortin MM Truitt RL Rimm AA Bach FH. Graft-Versus-Leukaemia Reactivity Induced by Alloimmunisation Without Augmentation of Graft-Versus-Host Reactivity. Nature (1979) 281:490–1. 10.1038/281490a0 386133

[27] Korngold R Sprent J. Lethal Graft-Versus-Host Disease After Bone Marrow Transplantation Across Minor Histocompatibility Barriers in Mice. Prevention by Removing Mature T Cells From Marrow. J Exp Med (1978) 148:1687–98. 10.1084/jem.148.6.1687 PMC2185109 363972

[28] Delorme EJ Alexander P. Treatment OF Primary Fibrosarcoma in the Rat With Immune Lymphocytes. Lancet (1964) 2:117–20. 10.1016/S0140-6736(64)90126-6 14160543

[29] Southam CM Brunschwig A Levin AG Dizon QS. Effect of Leukocytes on Transplantability of Human Cancer. Cancer (1966) 19:1743–53. 10.1002/1097-0142(196611)19:11<1743::AID-CNCR2820191143>3.0.CO;2-U 5925282

[30] Eberlein TJ Rosenstein M Rosenberg SA. Regression of a Disseminated Syngeneic Solid Tumor by Systemic Transfer of Lymphoid Cells Expanded in Interleukin 2. J Exp Med (1982) 156:385–97. 10.1084/jem.156.2.385 PMC2186754 6980254

[31] Mulé JJ Shu S Schwarz SL Rosenberg SA. Adoptive Immunotherapy of Established Pulmonary Metastases With LAK Cells and Recombinant Interleukin-2. Science (1984) 225:1487–9. 10.1126/science.6332379 6332379

[32] Donohue JH Rosenstein M Chang AE Lotze MT Robb RJ Rosenberg SA. The Systemic Administration of Purified Interleukin 2 Enhances the Ability of Sensitized Murine Lymphocytes to Cure a Disseminated Syngeneic Lymphoma. J Immunol (1984) 132:2123–8. 6607956

[33] Rosenberg SA Spiess P Lafreniere R. A New Approach to the Adoptive Immunotherapy of Cancer With Tumor-Infiltrating Lymphocytes. Science (1986) 233:1318–21. 10.1126/science.3489291 3489291

[34] Rosenberg SA Yannelli JR Yang JC Topalian SL Schwartzentruber DJ Weber JS. Treatment of Patients With Metastatic Melanoma With Autologous Tumor-Infiltrating Lymphocytes and Interleukin 2. J Natl Cancer Inst (1994) 86:1159–66. 10.1093/jnci/86.15.1159 8028037

[35] Rosenberg SA Yang JC Sherry RM Kammula US Hughes MS Phan GQ. Durable Complete Responses in Heavily Pretreated Patients With Metastatic Melanoma Using T-cell Transfer Immunotherapy. Clin Cancer Res (2011) 17:4550–7. 10.1158/1078-0432.CCR-11-0116 PMC3131487 21498393

[36] Yron I Wood TA Jr. Spiess PJ Rosenberg SA. In Vitro Growth of Murine T Cells. V. The Isolation and Growth of Lymphoid Cells Infiltrating Syngeneic Solid Tumors. J Immunol (1980) 125:238–45. 6966652

[37] Lotze MT Grimm EA Mazumder A Strausser JL Rosenberg SA. Lysis of Fresh and Cultured Autologous Tumor by Human Lymphocytes Cultured in T-cell Growth Factor. Cancer Res (1981) 41:4420–5. 6975652

[38] Grimm EA Mazumder A Zhang HZ Rosenberg SA. Lymphokine-Activated Killer Cell Phenomenon. Lysis of Natural Killer-Resistant Fresh Solid Tumor Cells by Interleukin 2-Activated Autologous Human Peripheral Blood Lymphocytes. J Exp Med (1982) 155:1823–41. 10.1084/jem.155.6.1823 PMC2186695 6176669

[39] Rosenstein M Yron I Kaufmann Y Rosenberg SA. Lymphokine-Activated Killer Cells: Lysis of Fresh Syngeneic Natural Killer-Resistant Murine Tumor Cells by Lymphocytes Cultured in Interleukin 2. Cancer Res (1984) 44:1946–53. 6608989

[40] Ettinghausen SE Lipford EH Mulé JJ Rosenberg SA. Recombinant Interleukin 2 Stimulates In Vivo Proliferation of Adoptively Transferred Lymphokine-Activated Killer (LAK) Cells. J Immunol (1985) 135:3623–35. 3900213

[41] Lafreniere R Rosenberg SA. Successful Immunotherapy of Murine Experimental Hepatic Metastases With Lymphokine-Activated Killer Cells and Recombinant Interleukin 2. Cancer Res (1985) 45:3735–41. 3893689

[42] Mazumder A Rosenberg SA. Successful Immunotherapy of Natural Killer-Resistant Established Pulmonary Melanoma Metastases by the Intravenous Adoptive Transfer of Syngeneic Lymphocytes Activated In Vitro by Interleukin 2. J Exp Med (1984) 159:495–507. 10.1084/jem.159.2.495 6141211 PMC2187217

[43] Mulé JJ Shu S Rosenberg SA. The Anti-Tumor Efficacy of Lymphokine-Activated Killer Cells and Recombinant Interleukin 2 In Vivo. J Immunol (1985) 135:646–52. 3889158

[44] Rosenberg SA Lotze MT Muul LM Leitman S Chang AE Ettinghausen SE. Observations on the Systemic Administration of Autologous Lymphokine-Activated Killer Cells and Recombinant Interleukin-2 to Patients With Metastatic Cancer. N Engl J Med (1985) 313:1485–92. 10.1056/NEJM198512053132327 3903508

[45] Rosenberg SA Mulé JJ Spiess PJ Reichert CM Schwarz SL. Regression of Established Pulmonary Metastases and Subcutaneous Tumor Mediated by the Systemic Administration of High-Dose Recombinant Interleukin 2. J Exp Med (1985) 161:1169–88. 10.1084/jem.161.5.1169 PMC2187617 3886826

[46] Muul LM Spiess PJ Director EP Rosenberg SA. Identification of Specific Cytolytic Immune Responses Against Autologous Tumor in Humans Bearing Malignant Melanoma. J Immunol (1987) 138:989–95. 3100623

[47] Topalian SL Muul LM Solomon D Rosenberg SA. Expansion of Human Tumor Infiltrating Lymphocytes for Use in Immunotherapy Trials. J Immunol Methods (1987) 102:127–41. 10.1016/S0022-1759(87)80018-2 3305708

[48] Dudley ME Wunderlich JR Robbins PF Yang JC Hwu P Schwartzentruber DJ. Cancer Regression and Autoimmunity in Patients After Clonal Repopulation With Antitumor Lymphocytes. Science (2002) 298:850–4. 10.1126/science.1076514 PMC1764179 12242449

[49] Rosenberg SA Aebersold P Cornetta K Kasid A Morgan RA Moen R. Gene Transfer Into Humans–Immunotherapy of Patients With Advanced Melanoma, Using Tumor-Infiltrating Lymphocytes Modified by Retroviral Gene Transduction. N Engl J Med (1990) 323:570–8. 10.1056/NEJM199008303230904 2381442

[50] Dudley ME Yang JC Sherry R Hughes MS Royal R Kammula U. Adoptive Cell Therapy for Patients With Metastatic Melanoma: Evaluation of Intensive Myeloablative Chemoradiation Preparative Regimens. J Clin Oncol (2008) 26:5233–9. 10.1200/JCO.2008.16.5449 PMC2652090 18809613

[51] Gattinoni L Finkelstein SE Klebanoff CA Antony PA Palmer DC Spiess PJ. Removal of Homeostatic Cytokine Sinks by Lymphodepletion Enhances the Efficacy of Adoptively Transferred Tumor-Specific CD8+ T Cells. J Exp Med (2005) 202:907–12. 10.1084/jem.20050732 PMC1397916 16203864

[52] Bronte V Wang M Overwijk WW Surman DR Pericle F Rosenberg SA. Apoptotic Death of CD8+ T Lymphocytes After Immunization: Induction of a Suppressive Population of Mac-1+/Gr-1+ Cells. J Immunol (1998) 161:5313–20. PMC2239007 9820504

[53] Yao X Ahmadzadeh M Lu YC Liewehr DJ Dudley ME Liu F. Levels of Peripheral CD4(+)FoxP3(+) Regulatory T Cells Are Negatively Associated With Clinical Response to Adoptive Immunotherapy of Human Cancer. Blood (2012) 119:5688–96. 10.1182/blood-2011-10-386482 PMC3382928 22555974

[54] Paulos CM Wrzesinski C Kaiser A Hinrichs CS Chieppa M Cassard L. Microbial Translocation Augments the Function of Adoptively Transferred Self/Tumor-Specific CD8+ T Cells Via TLR4 Signaling. J Clin Invest (2007) 117:2197–204. 10.1172/JCI32205 PMC1924500 17657310

[55] Turtle CJ Hanafi LA Berger C Gooley TA Cherian S Hudecek M. Cd19 CAR-T Cells of Defined CD4+:CD8+ Composition in Adult B Cell ALL Patients. J Clin Invest (2016) 126:2123–38. 10.1172/JCI85309 PMC4887159 27111235

[56] Tran E Turcotte S Gros A Robbins PF Lu YC Dudley ME. Cancer Immunotherapy Based on Mutation-Specific CD4+ T Cells in a Patient With Epithelial Cancer. Science (2014) 344:641–5. 10.1126/science.1251102 PMC6686185 24812403

[57] Yossef R Tran E Deniger DC Gros A Pasetto A Parkhurst MR. Enhanced Detection of Neoantigen-Reactive T Cells Targeting Unique and Shared Oncogenes for Personalized Cancer Immunotherapy. JCI Insight (2018) 3. 10.1172/jci.insight.122467 PMC6237474 30282837

[58] Veatch JR Lee SM Fitzgibbon M Chow IT Jesernig B Schmitt T. Tumor-Infiltrating BRAFV600E-Specific CD4+ T Cells Correlated With Complete Clinical Response in Melanoma. J Clin Invest (2018) 128:1563–8. 10.1172/JCI98689 PMC5873881 29360643

[59] Linnemann C van Buuren MM Bies L Verdegaal EM Schotte R Calis JJ. High-Throughput Epitope Discovery Reveals Frequent Recognition of Neo-Antigens by CD4+ T Cells in Human Melanoma. Nat Med (2015) 21:81–5. 10.1038/nm.3773 25531942

[60] Muranski P Borman ZA Kerkar SP Klebanoff CA Ji Y Sanchez-Perez L. Th17 Cells Are Long Lived and Retain a Stem Cell-Like Molecular Signature. Immunity (2011) 35:972–85. 10.1016/j.immuni.2011.09.019 PMC3246082 22177921

[61] Friedman KM Prieto PA Devillier LE Gross CA Yang JC Wunderlich JR. Tumor-Specific CD4+ Melanoma Tumor-Infiltrating Lymphocytes. J Immunother (2012) 35:400–8. 10.1097/CJI.0b013e31825898c5 PMC7412749 22576345

[62] Ben-Avi R Farhi R Ben-Nun A Gorodner M Greenberg E Markel G. Establishment of Adoptive Cell Therapy With Tumor Infiltrating Lymphocytes for Non-Small Cell Lung Cancer Patients. Cancer Immunol Immunother (2018) 67:1221–30. 10.1007/s00262-018-2174-4 PMC11028292 29845338

[63] Inozume T Hanada K Wang QJ Ahmadzadeh M Wunderlich JR Rosenberg SA. Selection of CD8+PD-1+ Lymphocytes in Fresh Human Melanomas Enriches for Tumor-Reactive T Cells. J Immunother (2010) 33:956–64. 10.1097/CJI.0b013e3181fad2b0 PMC2980947 20948441

[64] Ye Q Song DG Poussin M Yamamoto T Best A Li C. CD137 Accurately Identifies and Enriches for Naturally Occurring Tumor-Reactive T Cells in Tumor. Clin Cancer Res (2014) 20:44–55. 10.1158/1078-0432.CCR-13-0945 24045181 PMC3947326

[65] Seliktar-Ofir S Merhavi-Shoham E Itzhaki O Yunger S Markel G Schachter J. Selection of Shared and Neoantigen-Reactive T Cells for Adoptive Cell Therapy Based on CD137 Separation. Front Immunol (2017) 8:1211. 10.3389/fimmu.2017.01211 29067023 PMC5641376

[66] Webb JR Milne K Watson P Deleeuw RJ Nelson BH. Tumor-Infiltrating Lymphocytes Expressing the Tissue Resident Memory Marker CD103 Are Associated With Increased Survival in High-Grade Serous Ovarian Cancer. Clin Cancer Res (2014) 20:434–44. 10.1158/1078-0432.CCR-13-1877 24190978

[67] Malik BT Byrne KT Vella JL Zhang P Shabaneh TB Steinberg SM. Resident Memory T Cells in the Skin Mediate Durable Immunity to Melanoma. Sci Immunol (2017) 2. 10.1126/sciimmunol.aam6346 PMC5525335 28738020

[68] Duhen T Duhen R Montler R Moses J Moudgil T de Miranda NF. Co-Expression of CD39 and CD103 Identifies Tumor-Reactive CD8 T Cells in Human Solid Tumors. Nat Commun (2018) 9:2724. 10.1038/s41467-018-05072-0 30006565 PMC6045647

[69] Hinrichs CS Borman ZA Gattinoni L Yu Z Burns WR Huang J. Human Effector CD8+ T Cells Derived From Naive Rather Than Memory Subsets Possess Superior Traits for Adoptive Immunotherapy. Blood (2011) 117:808–14. 10.1182/blood-2010-05-286286 PMC3035075 20971955

[70] Miller BC Sen DR Al Abosy R Bi K Virkud YV LaFleur MW. Subsets of Exhausted CD8(+) T Cells Differentially Mediate Tumor Control and Respond to Checkpoint Blockade. Nat Immunol (2019) 20:326–36. 10.1038/s41590-019-0312-6 PMC6673650 30778252

[71] Siddiqui I Schaeuble K Chennupati V Fuertes Marraco SA Calderon-Copete S Ferreira DP. Intratumoral Tcf1(+)PD-1(+)CD8(+) T Cells With Stem-like Properties Promote Tumor Control in Response to Vaccination and Checkpoint Blockade Immunotherapy. Immunity (2019) 50:195–211.e110. 10.1016/j.immuni.2018.12.021 30635237

[72] Martinez-Usatorre A Carmona SJ Godfroid C Yacoub Maroun C Labiano S Romero P. Enhanced Phenotype Definition for Precision Isolation of Precursor Exhausted Tumor-Infiltrating Cd8 T Cells. Front Immunol (2020) 11:340. 10.3389/fimmu.2020.00340 32174925 PMC7056729

[73] Krishna S Lowery FJ Copeland AR Bahadiroglu E Mukherjee R Jia L. Stem-Like CD8 T Cells Mediate Response of Adoptive Cell Immunotherapy Against Human Cancer. Science (2020) 370:1328–34. 10.1126/science.abb9847 PMC8883579 33303615

[74] Bobisse S Genolet R Roberti A Tanyi JL Racle J Stevenson BJ. Sensitive and Frequent Identification of High Avidity Neo-Epitope Specific CD8 (+) T Cells in Immunotherapy-Naive Ovarian Cancer. Nat Commun (2018) 9:1092. 10.1038/s41467-018-03301-0 29545564 PMC5854609

[75] Sukumar M Liu J Mehta GU Patel SJ Roychoudhuri R Crompton JG. Mitochondrial Membrane Potential Identifies Cells With Enhanced Stemness for Cellular Therapy. Cell Metab (2016) 23:63–76. 10.1016/j.cmet.2015.11.002 26674251 PMC4747432

[76] Lu YC Zheng Z Robbins PF Tran E Prickett TD Gartner JJ. An Efficient Single-Cell RNA-Seq Approach to Identify Neoantigen-Specific T Cell Receptors. Mol Ther (2018) 26:379–89. 10.1016/j.ymthe.2017.10.018 PMC5835023 29174843

[77] Schumacher TN Scheper W Kvistborg P. Cancer Neoantigens. Annu Rev Immunol (2019) 37:173–200. 10.1146/annurev-immunol-042617-053402 30550719

[78] Schumacher TN Schreiber RD. Neoantigens in Cancer Immunotherapy. Science (2015) 348:69–74. 10.1126/science.aaa4971 25838375

[79] Snyder A Makarov V Merghoub T Yuan J Zaretsky JM Desrichard A. Genetic Basis for Clinical Response to CTLA-4 Blockade in Melanoma. N Engl J Med (2014) 371:2189–99. 10.1056/NEJMoa1406498 PMC4315319 25409260

[80] Lennerz V Fatho M Gentilini C Frye RA Lifke A Ferel D. The Response of Autologous T Cells to a Human Melanoma Is Dominated by Mutated Neoantigens. Proc Natl Acad Sci USA (2005) 102:16013–8. 10.1073/pnas.0500090102 PMC1266037 16247014

[81] Rizvi NA Hellmann MD Snyder A Kvistborg P Makarov V Havel JJ. Cancer Immunology. Mutational Landscape Determines Sensitivity to PD-1 Blockade in Non-Small Cell Lung Cancer. Science (2015) 348:124–8. 10.1126/science.aaa1348 PMC4993154 25765070

[82] Huang J El-Gamil M Dudley ME Li YF Rosenberg SA Robbins PF. T Cells Associated With Tumor Regression Recognize Frameshifted Products of the CDKN2A Tumor Suppressor Gene Locus and a Mutated HLA Class I Gene Product. J Immunol (2004) 172:6057–64. 10.4049/jimmunol.172.10.6057 PMC2305724 15128789

[83] Zhou J Dudley ME Rosenberg SA Robbins PF. Persistence of Multiple Tumor-Specific T-Cell Clones Is Associated With Complete Tumor Regression in a Melanoma Patient Receiving Adoptive Cell Transfer Therapy. J Immunother (2005) 28:53–62. 10.1097/00002371-200501000-00007 15614045 PMC2175172

[84] Lu YC Yao X Li YF El-Gamil M Dudley ME Yang JC. Mutated PPP1R3B Is Recognized by T Cells Used to Treat a Melanoma Patient Who Experienced a Durable Complete Tumor Regression. J Immunol (2013) 190:6034–42. 10.4049/jimmunol.1202830 PMC3679246 23690473

[85] Robbins PF Lu YC El-Gamil M Li YF Gross C Gartner J. Mining Exomic Sequencing Data to Identify Mutated Antigens Recognized by Adoptively Transferred Tumor-Reactive T Cells. Nat Med (2013) 19:747–52. 10.1038/nm.3161 PMC3757932 23644516

[86] Lu YC Yao X Crystal JS Li YF El-Gamil M Gross C. Efficient Identification of Mutated Cancer Antigens Recognized by T Cells Associated With Durable Tumor Regressions. Clin Cancer Res (2014) 20:3401–10. 10.1158/1078-0432.CCR-14-0433 PMC4083471 24987109

[87] Prickett TD Crystal JS Cohen CJ Pasetto A Parkhurst MR Gartner JJ. Durable Complete Response From Metastatic Melanoma After Transfer of Autologous T Cells Recognizing 10 Mutated Tumor Antigens. Cancer Immunol Res (2016) 4:669–78. 10.1158/2326-6066.CIR-15-0215 PMC4970903 27312342

[88] Stevanović S Pasetto A Helman SR Gartner JJ Prickett TD Howie B. Landscape of Immunogenic Tumor Antigens in Successful Immunotherapy of Virally Induced Epithelial Cancer. Science (2017) 356:200–5. 10.1126/science.aak9510 PMC6295311 28408606

[89] Li Q Ding ZY. The Ways of Isolating Neoantigen-Specific T Cells. Front Oncol (2020) 10:1347. 10.3389/fonc.2020.01347 32850430 PMC7431921

[90] Matsushita H Vesely MD Koboldt DC Rickert CG Uppaluri R Magrini VJ. Cancer Exome Analysis Reveals a T-cell-dependent Mechanism of Cancer Immunoediting. Nature (2012) 482:400–4. 10.1038/nature10755 PMC3874809 22318521

[91] Castle JC Kreiter S Diekmann J Löwer M van de Roemer N de Graaf J. Exploiting the Mutanome for Tumor Vaccination. Cancer Res (2012) 72:1081–91. 10.1158/0008-5472.CAN-11-3722 22237626

[92] Zamora AE Crawford JC Allen EK Guo XJ Bakke J Carter RA. Pediatric Patients With Acute Lymphoblastic Leukemia Generate Abundant and Functional Neoantigen-Specific CD8(+) T Cell Responses. Sci Transl Med (2019) 11. 10.1126/scitranslmed.aat8549 PMC7020562 31243155

[93] Tran E Robbins PF Lu YC Prickett TD Gartner JJ Jia L. T-Cell Transfer Therapy Targeting Mutant KRAS in Cancer. N Engl J Med (2016) 375:2255–62. 10.1056/NEJMoa1609279 PMC5178827 27959684

[94] Martin SD Wick DA Nielsen JS Little N Holt RA Nelson BH. A Library-Based Screening Method Identifies Neoantigen-Reactive T Cells in Peripheral Blood Prior to Relapse of Ovarian Cancer. Oncoimmunology (2017) 7:e1371895. 10.1080/2162402X.2017.1371895 29296522 PMC5739566

[95] Zacharakis N Chinnasamy H Black M Xu H Lu YC Zheng Z. Immune Recognition of Somatic Mutations Leading to Complete Durable Regression in Metastatic Breast Cancer. Nat Med (2018) 24:724–30. 10.1038/s41591-018-0040-8 PMC6348479 29867227

[96] Tran E Ahmadzadeh M Lu YC Gros A Turcotte S Robbins PF. Immunogenicity of Somatic Mutations in Human Gastrointestinal Cancers. Science (2015) 350:1387–90. 10.1126/science.aad1253 PMC7445892 26516200

[97] Rath JA Arber C. Engineering Strategies to Enhance TCR-Based Adoptive T Cell Therapy. Cells (2020) 9:1485. 10.3390/cells9061485 PMC7349724 32570906

[98] Grimes JM Carvajal RD Muranski P. Cellular Therapy for the Treatment of Solid Tumors. Transfus Apher Sci (2021) 60(1):103056. 10.1016/j.transci.2021.103056 33478888

[99] Verdegaal EM Visser M Ramwadhdoebé TH van der Minne CE van Steijn JA Kapiteijn E. Successful Treatment of Metastatic Melanoma by Adoptive Transfer of Blood-Derived Polyclonal Tumor-Specific CD4+ and CD8+ T Cells in Combination With Low-Dose Interferon-Alpha. Cancer Immunol Immunother (2011) 60:953–63. 10.1007/s00262-011-1004-8 PMC3119331 21431917

[100] Gros A Parkhurst MR Tran E Pasetto A Robbins PF Ilyas S. Prospective Identification of Neoantigen-Specific Lymphocytes in the Peripheral Blood of Melanoma Patients. Nat Med (2016) 22:433–8. 10.1038/nm.4051 PMC7446107 26901407

[101] Gros A Robbins PF Yao X Li YF Turcotte S Tran E. PD-1 Identifies the Patient-Specific CD8⁺ Tumor-Reactive Repertoire Infiltrating Human Tumors. J Clin Invest (2014) 124:2246–59. 10.1172/JCI73639 PMC4001555 24667641

[102] Simpson AJ Caballero OL Jungbluth A Chen YT Old LJ. Cancer/Testis Antigens, Gametogenesis and Cancer. Nat Rev Cancer (2005) 5:615–25. 10.1038/nrc1669 16034368

[103] Caballero OL Chen YT. Cancer/Testis (CT) Antigens: Potential Targets for Immunotherapy. Cancer Sci (2009) 100:2014–21. 10.1111/j.1349-7006.2009.01303.x PMC11158245 19719775

[104] van der Woude LL Gorris MAJ Halilovic A Figdor CG de Vries IJM. Migrating Into the Tumor: A Roadmap for T Cells. Trends Cancer (2017) 3:797–808. 10.1016/j.trecan.2017.09.006 29120755

[105] Whitehurst AW. Cause and Consequence of Cancer/Testis Antigen Activation in Cancer. Annu Rev Pharmacol Toxicol (2014) 54:251–72. 10.1146/annurev-pharmtox-011112-140326 24160706

[106] Robbins PF Morgan RA Feldman SA Yang JC Sherry RM Dudley ME. Tumor Regression in Patients With Metastatic Synovial Cell Sarcoma and Melanoma Using Genetically Engineered Lymphocytes Reactive With NY-ESO-1. J Clin Oncol (2011) 29:917–24. 10.1200/JCO.2010.32.2537 PMC3068063 21282551

[107] Morgan RA Chinnasamy N Abate-Daga D Gros A Robbins PF Zheng Z. Cancer Regression and Neurological Toxicity Following Anti-MAGE-A3 TCR Gene Therapy. J Immunother (2013) 36:133–51. 10.1097/CJI.0b013e3182829903 PMC3581823 23377668

[108] Johnson LA Morgan RA Dudley ME Cassard L Yang JC Hughes MS. Gene Therapy With Human and Mouse T-cell Receptors Mediates Cancer Regression and Targets Normal Tissues Expressing Cognate Antigen. Blood (2009) 114:535–46. 10.1182/blood-2009-03-211714 PMC2929689 19451549

[109] Leko V Rosenberg SA. Identifying and Targeting Human Tumor Antigens for T Cell-Based Immunotherapy of Solid Tumors. Cancer Cell (2020) 38:454–72. 10.1016/j.ccell.2020.07.013 PMC7737225 32822573

[110] Dafni U Michielin O Lluesma SM Tsourti Z Polydoropoulou V Karlis D. Efficacy of Adoptive Therapy With Tumor-Infiltrating Lymphocytes and Recombinant Interleukin-2 in Advanced Cutaneous Melanoma: A Systematic Review and Meta-Analysis. Ann Oncol (2019) 30:1902–13. 10.1093/annonc/mdz398 31566658

[111] Sarnaik A Khushalani NI Chesney JA Lewis KD Medina TM Kluger HM. Long-Term Follow Up of Lifileucel (LN-144) Cryopreserved Autologous Tumor Infiltrating Lymphocyte Therapy in Patients With Advanced Melanoma Progressed on Multiple Prior Therapies. J Clin Oncol (2020) 38:10006–6. 10.1200/JCO.2020.38.15_suppl.10006

[112] Jazaeri AA Zsiros E Amaria RN Artz AS Edwards RP Wenham RM. Safety and Efficacy of Adoptive Cell Transfer Using Autologous Tumor Infiltrating Lymphocytes (LN-145) for Treatment of Recurrent, Metastatic, or Persistent Cervical Carcinoma. J Clin Oncol (2019) 37:2538–8. 10.1200/JCO.2019.37.15_suppl.2538

[113] Creelan B Wang C Teer J Toloza E Mullinax J Yao J. Abstract CT056: Durable Complete Responses to Adoptive Cell Transfer Using Tumor Infiltrating Lymphocytes (TIL) in Non-Small Cell Lung Cancer (NSCLC): A Phase I Trial. Cancer Res (2020) 80:CT056–6. 10.1158/1538-7445.AM2020-CT056

[114] Pardoll DM. The Blockade of Immune Checkpoints in Cancer Immunotherapy. Nat Rev Cancer (2012) 12:252–64. 10.1038/nrc3239 PMC4856023 22437870

[115] Sarnaik A Khushalani NI Chesney JA Kluger HM Curti BD Lewis KD. Safety and Efficacy of Cryopreserved Autologous Tumor Infiltrating Lymphocyte Therapy (LN-144, Lifileucel) in Advanced Metastatic Melanoma Patients Who Progressed on Multiple Prior Therapies Including Anti-PD-1. J Clin Oncol (2019) 37:2518–8. 10.1200/JCO.2019.37.15_suppl.2518

[116] Sarnaik A Kluger HM Chesney JA Sethuraman J Veerapathran A Simpson-Abelson M. Efficacy of Single Administration of Tumor-Infiltrating Lymphocytes (TIL) in Heavily Pretreated Patients With Metastatic Melanoma Following Checkpoint Therapy. J Clin Oncol (2017) 35:3045–5. 10.1200/JCO.2017.35.15_suppl.3045

[117] Zimmer L Apuri S Eroglu Z Kottschade LA Forschner A Gutzmer R. Ipilimumab Alone or in Combination With Nivolumab After Progression on Anti-PD-1 Therapy in Advanced Melanoma. Eur J Cancer (2017) 75:47–55. 10.1016/j.ejca.2017.01.009 28214657

[118] Brahmer JR Drake CG Wollner I Powderly JD Picus J Sharfman WH. Phase I Study of Single-Agent Anti-Programmed Death-1 (MDX-1106) in Refractory Solid Tumors: Safety, Clinical Activity, Pharmacodynamics, and Immunologic Correlates. J Clin Oncol (2010) 28:3167–75. 10.1200/JCO.2009.26.7609 PMC4834717 20516446

[119] Cottrell TR Taube JM. PD-L1 and Emerging Biomarkers in Immune Checkpoint Blockade Therapy. Cancer J (2018) 24:41–6. 10.1097/PPO.0000000000000301 PMC5830145 29360727

[120] Kverneland AH Pedersen M Westergaard MCW Nielsen M Borch TH Olsen LR. Adoptive Cell Therapy in Combination With Checkpoint Inhibitors in Ovarian Cancer. Oncotarget (2020) 11:2092–105. 10.18632/oncotarget.27604 PMC7275789 32547707

[121] Wells DK van Buuren MM Dang KK Hubbard-Lucey VM Sheehan KCF Campbell KM. Key Parameters of Tumor Epitope Immunogenicity Revealed Through a Consortium Approach Improve Neoantigen Prediction. Cell (2020) 183:818–34.e813. 10.1016/j.cell.2020.09.015 33038342 PMC7652061

[122] Alexandrov LB Nik-Zainal S Wedge DC Aparicio SA Behjati S Biankin AV. Signatures of Mutational Processes in Human Cancer. Nature (2013) 500:415–21. 10.1038/nature12477 PMC3776390 23945592

[123] Chen DS Mellman I. Elements of Cancer Immunity and the Cancer-Immune Set Point. Nature (2017) 541:321–30. 10.1038/nature21349 28102259

[124] Kim JY Kronbichler A Eisenhut M Hong SH van der Vliet HJ Kang J. Tumor Mutational Burden and Efficacy of Immune Checkpoint Inhibitors: A Systematic Review and Meta-Analysis. Cancers (Basel) (2019) 11(11):1798. 10.3390/cancers11111798 PMC6895916 31731749

[125] Marabelle A Fakih M Lopez J Shah M Shapira-Frommer R Nakagawa K. Association of Tumour Mutational Burden With Outcomes in Patients With Advanced Solid Tumours Treated With Pembrolizumab: Prospective Biomarker Analysis of the Multicohort, Open-Label, Phase 2 KEYNOTE-158 Study. Lancet Oncol (2020) 21(10):1353–65. 10.1016/S1470-2045(20)30445-9 32919526

[126] Pecina-Slaus N Kafka A Salamon I Bukovac A. Mismatch Repair Pathway, Genome Stability and Cancer. Front Mol Biosci (2020) 7:122. 10.3389/fmolb.2020.00122 32671096 PMC7332687

[127] Le DT Uram JN Wang H Bartlett BR Kemberling H Eyring AD. PD-1 Blockade in Tumors With Mismatch-Repair Deficiency. N Engl J Med (2015) 372:2509–20. 10.1056/NEJMoa1500596 PMC4481136 26028255

[128] Andre T Shiu K-K Kim TW Jensen BV Jensen LH Punt CJA. Pembrolizumab Versus Chemotherapy for Microsatellite Instability-High/Mismatch Repair Deficient Metastatic Colorectal Cancer: The Phase 3 KEYNOTE-177 Study. J Clin Oncol (2020) 38:LBA4–4. 10.1200/JCO.2020.38.18_suppl.LBA4

[129] Yin H Guo W Sun X Li R Feng C Tan Y. TILs and Anti-PD1 Therapy: An Alternative Combination Therapy for PDL1 Negative Metastatic Cervical Cancer. J Immunol Res (2020) 2020:8345235. 10.1155/2020/8345235 32964058 PMC7492938

[130] Stevanovic S Helman SR Wunderlich JR Langhan MM Doran SL Kwong MLM. A Phase II Study of Tumor-infiltrating Lymphocyte Therapy for Human Papillomavirus-Associated Epithelial Cancers. Clin Cancer Res (2019) 25:1486–93. 10.1158/1078-0432.CCR-18-2722 PMC6397671 30518633

[131] Gao P Lazare C Cao C Meng Y Wu P Zhi W. Immune Checkpoint Inhibitors in the Treatment of Virus-Associated Cancers. J Hematol Oncol (2019) 12:58. 10.1186/s13045-019-0743-4 31182108 PMC6558794

[132] Seiwert TY Burtness B Mehra R Weiss J Berger R Eder JP. Safety and Clinical Activity of Pembrolizumab for Treatment of Recurrent or Metastatic Squamous Cell Carcinoma of the Head and Neck (KEYNOTE-012): An Open-Label, Multicentre, Phase 1b Trial. Lancet Oncol (2016) 17:956–65. 10.1016/S1470-2045(16)30066-3 27247226

[133] Zandberg DP Algazi AP Jimeno A Good JS Fayette J Bouganim N. Durvalumab for Recurrent or Metastatic Head and Neck Squamous Cell Carcinoma: Results From a Single-Arm, Phase II Study in Patients With ≥25% Tumour Cell PD-L1 Expression Who Have Progressed on Platinum-Based Chemotherapy. Eur J Cancer (2019) 107:142–52. 10.1016/j.ejca.2018.11.015 30576970

[134] Kim ST Cristescu R Bass AJ Kim KM Odegaard JI Kim K. Comprehensive Molecular Characterization of Clinical Responses to PD-1 Inhibition in Metastatic Gastric Cancer. Nat Med (2018) 24:1449–58. 10.1038/s41591-018-0101-z 30013197

[135] Cook MR Kim C. Safety and Efficacy of Immune Checkpoint Inhibitor Therapy in Patients With HIV Infection and Advanced-Stage Cancer: A Systematic Review. JAMA Oncol (2019) 5:1049–54. 10.1001/jamaoncol.2018.6737 30730549

[136] Stevanovic S Draper LM Langhan MM Campbell TE Kwong ML Wunderlich JR. Complete Regression of Metastatic Cervical Cancer After Treatment With Human Papillomavirus-Targeted Tumor-Infiltrating T Cells. J Clin Oncol (2015) 33:1543–50. 10.1200/JCO.2014.58.9093 PMC4417725 25823737

[137] Ayers M Lunceford J Nebozhyn M Murphy E Loboda A Kaufman DR. IFN-Gamma-Related mRNA Profile Predicts Clinical Response to PD-1 Blockade. J Clin Invest (2017) 127:2930–40. 10.1172/JCI91190 PMC5531419 28650338

[138] Cristescu R Mogg R Ayers M Albright A Murphy E Yearley J. Pan-Tumor Genomic Biomarkers for PD-1 Checkpoint Blockade-Based Immunotherapy. Science (2018) 362(6411). 10.1126/science.aar3593 PMC6718162 30309915

[139] Wolf B Zimmermann S Arber C Irving M Trueb L Coukos G. Safety and Tolerability of Adoptive Cell Therapy in Cancer. Drug Saf (2019) 42:315–34. 10.1007/s40264-018-0779-3 30649750

[140] Dudley ME Wunderlich JR Yang JC Sherry RM Topalian SL Restifo NP. Adoptive Cell Transfer Therapy Following Non-Myeloablative But Lymphodepleting Chemotherapy for the Treatment of Patients With Refractory Metastatic Melanoma. J Clin Oncol (2005) 23:2346–57. 10.1200/JCO.2005.00.240 PMC1475951 15800326

[141] Besser MJ Shapira-Frommer R Itzhaki O Treves AJ Zippel DB Levy D. Adoptive Transfer of Tumor-Infiltrating Lymphocytes in Patients With Metastatic Melanoma: Intent-to-Treat Analysis and Efficacy After Failure to Prior Immunotherapies. Clin Cancer Res (2013) 19:4792–800. 10.1158/1078-0432.CCR-13-0380 23690483

[142] Dudley ME Gross CA Langhan MM Garcia MR Sherry RM Yang JC. CD8+ Enriched “Young” Tumor Infiltrating Lymphocytes Can Mediate Regression of Metastatic Melanoma. Clin Cancer Res (2010) 16:6122–31. 10.1158/1078-0432.CCR-10-1297 PMC2978753 20668005

[143] Dudley ME Gross CA Somerville RP Hong Y Schaub NP Rosati SF. Randomized Selection Design Trial Evaluating CD8+-Enriched Versus Unselected Tumor-Infiltrating Lymphocytes for Adoptive Cell Therapy for Patients With Melanoma. J Clin Oncol (2013) 31:2152–9. 10.1200/JCO.2012.46.6441 PMC3731980 23650429

[144] Hong JJ Rosenberg SA Dudley ME Yang JC White DE Butman JA. Successful Treatment of Melanoma Brain Metastases With Adoptive Cell Therapy. Clin Cancer Res (2010) 16:4892–8. 10.1158/1078-0432.CCR-10-1507 PMC6291850 20719934

[145] Radvanyi LG Bernatchez C Zhang M Fox PS Miller P Chacon J. Specific Lymphocyte Subsets Predict Response to Adoptive Cell Therapy Using Expanded Autologous Tumor-Infiltrating Lymphocytes in Metastatic Melanoma Patients. Clin Cancer Res (2012) 18:6758–70. 10.1158/1078-0432.CCR-12-1177 PMC3525747 23032743

[146] Rosenberg SA Packard BS Aebersold PM Solomon D Topalian SL Toy ST. Use of Tumor-Infiltrating Lymphocytes and Interleukin-2 in the Immunotherapy of Patients With Metastatic Melanoma. A Preliminary Report. N Engl J Med (1988) 319:1676–80. 10.1056/NEJM198812223192527 3264384

[147] Haanen JBAG Carbonnel F Robert C Kerr KM Peters S Larkin J. ESMO Guidelines Committee. Management of toxicities from immunotherapy: ESMOClinical Practice Guidelines for Diagnosis, Treatment and Follow-Up. Ann Oncol (2017) 28(suppl_4):iv119–42. 10.1093/annonc/mdx225 28881921

[148] Topalian SL Solomon D Avis FP Chang AE Freerksen DL Linehan WM. Immunotherapy of Patients With Advanced Cancer Using Tumor-Infiltrating Lymphocytes and Recombinant interleukin-2: A Pilot Study. J Clin Oncol (1988) 6:839–53. 10.1200/JCO.1988.6.5.839 3259261

[149] Yeh S Karne NK Kerkar SP Heller CK Palmer DC Johnson LA. Ocular and Systemic Autoimmunity After Successful Tumor-Infiltrating Lymphocyte Immunotherapy for Recurrent, Metastatic Melanoma. Ophthalmology (2009) 116:981–9.e981. 10.1016/j.ophtha.2008.12.004 19410956 PMC2715843

[150] Dudley ME Wunderlich JR Yang JC Hwu P Schwartzentruber DJ Topalian SL. A Phase I Study of Nonmyeloablative Chemotherapy and Adoptive Transfer of Autologous Tumor Antigen-Specific T Lymphocytes in Patients With Metastatic Melanoma. J Immunother (2002) 25:243–51. 10.1097/00002371-200205000-00007 PMC2413438 12000866

[151] Dutcher JP Schwartzentruber DJ Kaufman HL Agarwala SS Tarhini AA Lowder JN. High Dose Interleukin-2 (Aldesleukin) - Expert Consensus on Best Management Practices-2014. J Immunother Cancer (2014) 2:26. 10.1186/s40425-014-0026-0 31546315 PMC6889624

[152] Atkins MB Lotze MT Dutcher JP Fisher RI Weiss G Margolin K. High-Dose Recombinant Interleukin 2 Therapy for Patients With Metastatic Melanoma: Analysis of 270 Patients Treated Between 1985 and 1993. J Clin Oncol (1999) 17:2105–16. 10.1200/JCO.1999.17.7.2105 10561265

[153] Schwartz RN Stover L Dutcher JP. Managing Toxicities of High-Dose Interleukin-2. Oncol (Williston Park) (2002) 16:11–20. 12469935

[154] Schwartzentruber DJ. Guidelines for the Safe Administration of High-Dose Interleukin-2. J Immunother (2001) 24:287–93. 10.1097/00002371-200107000-00004 11565830

